# The effect of *Azorhizobium caulinodans* ORS571 and γ-aminobutyric acid on salt tolerance of *Sesbania rostrata*

**DOI:** 10.3389/fpls.2022.926850

**Published:** 2022-08-05

**Authors:** Yanan Liu, Xiaolin Liu, Xiaoyan Dong, Jiaming Yan, Zhihong Xie, Yongming Luo

**Affiliations:** ^1^CAS Key Laboratory of Coastal Environmental Processes and Ecological Remediation, Yantai Institute of Coastal Zone Research, Chinese Academy of Sciences, Yantai, China; ^2^University of Chinese Academy of Sciences, Beijing, China; ^3^National Engineering Research Center for Efficient Utilization of Soil and Fertilizer Resources, College of Resources and Environment of Shandong Agricultural University, Taian, China; ^4^CAS Key Laboratory of Soil Environment and Pollution Remediation, Institute of Soil Science, Chinese Academy of Sciences, Nanjing, China

**Keywords:** *Sesbania rostrata*, *Azorhizobium caulinodans* ORS571, γ-aminobutyric acid, NaCl stress, antioxidant enzymes, chlorophyll

## Abstract

Salt stress seriously affects plant growth and crop yield, and has become an important factor that threatens the soil quality worldwide. In recent years, the cultivation of salt-tolerant plants such as *Sesbania rostrata* has a positive effect on improving coastal saline-alkali land. Microbial inoculation and GABA addition have been shown to enhance the plant tolerance in response to the abiotic stresses, but studies in green manure crops and the revelation of related mechanisms are not clear. In this study, the effects of inoculation with *Azorhizobium caulinodans* ORS571 and exogenous addition of γ-Aminobutyric Acid (GABA; 200 mg·L^−1^) on the growth and development of *S. rostrata* under salt stress were investigated using potting experiments of vermiculite. The results showed that inoculation with ORS571 significantly increased the plant height, biomass, chlorophyll content, proline content (PRO), catalase (CAT) activity, and superoxide dismutase (SOD) activity of *S. rostrata* and reduced the malondialdehyde (MDA) level of leaves. The exogenous addition of GABA also increased the height, biomass, and CAT activity and reduced the MDA and PRO level of leaves. In addition, exogenous addition of GABA still had a certain improvement on the CAT activity and chlorophyll content of the ORS571-*S. rostrata* symbiotic system. In conclusion, ORS571 inoculation and GABA application have a positive effect on improving the salt stress tolerance in *S. rostrata*, which are closely associated with increasing chlorophyll synthesis and antioxidant enzyme activity and changing the amino acid content. Therefore, it can be used as a potential biological measure to improve the saline-alkali land.

## Background

Salt stress is a major constraint on the performance of plant, which can reduce the growth and yield of crop (Chen et al., 2016). It was estimated that one-twentieth of the global total area and one-fifth of the irrigated soil are affected by salinity (Morton et al., 2019). Therefore, the management and restoration of saline lands is crucial. In recent years, the bioremediation method represented by planting salt-tolerant plants has been used to improve saline-alkali soil due to its low cost, wide application, and good effect (Xia et al., 2019; Cui et al., 2021). *Sesbania rostrata*, a semi-aquatic legume indigenous to the Sahel region of Africa, is moderately salt tolerant and may be utilized as forage crop and green manure on saline land (Mahmood, 1998). Previous studies have demonstrated that *S. rostrata* can adapt to a high salinity environment by fixing and transferring salt, meanwhile regulating the osmotic response and antioxidant enzyme activity (Jungklang et al., 2003, 2004). However, with the increase of salt concentration, the effect of *S. rostrata* on the improvement of saline soil is still limited.

The plant growth-promoting bacteria (PGPB) are able to stimulate plant growth directly or indirectly, thus establishing a good ecological relationship with plants (Jiménez-Mejía et al., 2022). The application of PGPB is now considered as one of the technologies for the development of sustainable agriculture (Santoyo et al., 2021). It has been demonstrated that PGPB can protect plants from salt stress by expressing 1-aminocyclopropane-1-carboxylate (ACC) deaminase or promoting the nutrient uptake of plant, increasing antioxidant enzyme activity, etc. (Suarez et al., 2015; Kumar et al., 2020; Jiménez-Mejía et al., 2022). A PGPB strain of *Azorhizobium caulinodans* ORS571, isolated from stem nodules of *S. rostrata*, has the dual capacity to fix nitrogen both as free-living organism and in a symbiotic interaction with *S. rostrata* (Lee et al., 2008). Several studies have characterized the involvement of proteins such as CheY1, CheY2, TlpA1, and CheZ in ORS571 in the processes of chemotaxis and host colonization (Liu et al., 2017, 2018, 2020). Studies have shown that inoculation of ORS571 could improve the germination rate of wheat seeds under drought stress and enhance the drought tolerance of wheat seedings (Liu et al., 2012). Another recent study showed that co-inoculation of *A. caulinodans* ORS571 and *Piriformospora indica* enhanced the tolerance of tomato to salt stress (Xu et al., 2022). However, it is not clear about the effect of ORS571 on salt stress tolerance in the host *S. rostrata*.

To address a series of physiological hazards associated with salinity, many chemicals such as hydrogen sulfide, nitric oxide (NO), and phytohormones have been found to improve the salt tolerance of plants. Studies have shown that exogenous application of the NO donor sodium nitroprusside (SNP) improved the salt tolerance of a variety of crops (Goyal et al., 2021). Hydrogen sulfide could also alleviate plant salt stress by increasing the K/Na ratio, protecting the photosynthetic apparatus, and enhancing the antioxidant system (Hasanuzzaman et al., 2020; Goyal et al., 2021). Osmoregulation mediated by phytohormone such as abscisic acid (ABA) has been found to play an important role in salt stress. In addition, the alleviating effect of γ-aminobutyric acid on salt stress has been gradually discovered. γ-aminobutyric acid (GABA), a natural non-protein amino acid, is widely distributed in plants, animals, and microorganisms (Steward et al., 1949; Roberts and Frankel, 1950; Siragusa et al., 2007). The biosynthesis of GABA is mainly executed through the γ-aminobutyric acid shunt, which contains three key enzymes: glutamate decarboxylase (GAD), GABA transaminase (GABA-T), and succinic semialdehyde dehydrogenase (SSADH; Sarasa et al., 2020). GABA acts as an important inhibitory neurotransmitter involved in many life processes of animals and has been used in the food and pharmaceutical industries (Roberts and Eidelberg, 1960; Kim et al., 2009). It can also improve the acid stress tolerance and spore germination rate of microorganisms, and may be involved in the regulation of bacterial biofilms and quorum sensing (Chevrot et al., 2006; Dagorn et al., 2013). Many studies have shown that GABA can act as a signaling molecule or metabolite to promote plant growth and alleviate abiotic stress, and GABA accumulation increased rapidly in response to adverse factors in rice and tea (Mei et al., 2016; Sheteiwy et al., 2019). Furthermore, it was shown that exogenous addition of GABA was able to alleviate growth inhibition under salt stress by inducing an increase in endogenous GABA as well as stress-resistant substances in barley and tomato seedlings (Ma et al., 2018; Wu et al., 2020). The current research mainly focused on the alleviation of salt stress in crops by the addition of exogenous GABA, and few studies have been carried out on the green manure crop. Interestingly, the latest study revealed that the combined application of PGPB and NO promotes salt stress tolerance in sugarcane plants, and the effect is better than that of application alone (Sharma et al., 2021). The combined application of multiple measures may play a better role in alleviating salt stress.

It is widely known that the salt-tolerance mechanisms involved in plants mainly include antioxidant enzyme activation, osmotic stress, ion toxicity, and hormone modulation (Numan et al., 2018). Salt stress can cause oxidative stress in plants. Reactive oxygen species (ROS) have a powerful oxidative capacity, which can cause membrane damage and irreversible metabolic dysfunction (Dhindsa et al., 1982). SOD and CAT are important antioxidant enzymes that protect plants from ROS damage. In addition, excessive ROS under high salt stress leads to an increase in the lipid peroxidation product MDA, the level of which can be considered as an indicator of salt stress in plants (Liang et al., 2018). Studies have shown that PRO as an osmoprotectant plays a positive role in the salt stress of plants (Ashraf and Foolad, 2007). The regulation of ion homeostasis in *Arabidopsis* is also closely related to plant salt tolerance (Deinlein et al., 2014). The ability of plants to maintain a high cytoplasmic K/Na ratio is one of the key determinants of salt tolerance in plants (Maathuis and Amtmann, 1999).

As mentioned above, the adaptation to salt stress of *S. rostrata* is limited, and how to improve its salt tolerance has become a question worth considering. In this study, the degree of damage to *S. rostrata* under salt stress was investigated, and the effects of inoculation with ORS571 on the growth and salt tolerance of *S. rostrata* were also analyzed. In addition, we further revealed the effect of exogenous addition of GABA on the salt stress tolerance of *S. rostrata* and ORS571-*S. rostrata* symbiotic system, and explored the related mechanisms.

## Materials and methods

### Plant materials and cultivation

*S. rostrata* seeds were surface sterilized by treating with concentrated sulfuric acid for 30 min, and then washed three times with sterile water. The seeds were placed in a sterile plate at 37°C in the dark to promote germination, and washed every 12 h to remove the seed secretions. After 36–48 h, there were ready to be planted when the roots grew to 2–4 cm. Germinated seeds were transplanted to Leonard jars filled with sterile vermiculite (premixed with low nitrogen nutrient solution) after appropriate treatment (Jiang et al., 2016). Plants were grown in a greenhouse at 28°C with a 12 h light photoperiod. Six replicates were set up in each experimental group, and one plant was planted in the Leonard jars for each replicate.

#### Inoculation treatment

ORS571 was activated to stationary phase with TY liquid medium (tryptone 5 g·L^−1^, yeast extract 3 g·L^−1^, and calcium chloride dihydrate 0.88 g·L^−1^, pH 7.4), and the OD_600_ was adjusted to 1.0. Take 10 ml of bacterial culture to immerse the seeds for 1 h for the inoculation test group, and take the same amount of medium to immerse the seeds for 1 h for the non-inoculation test group.

#### Salt treatment (Na)

In the pre-test, 100 and 200 mM NaCl were screened for the optimum concentration by consulting the literature (Mahmood, 1998). The distilled water in the lower layer of Leonard jars was replaced by three sets of NaCl stress gradients, 0, 100, and 200 mM, respectively. NaCl stress was performed when the seedlings grew to the second compound leaf (about 16 days), and the plants were harvested after another 20 days.

#### GABA treatment

200 mg·L^−1^ GABA (Nanning Harworld Biological Technology) was added to Leonard jars with NaCl.

### Determination of height above ground and number of nodules

The roots were washed and the surface water was absorbed after the plants were taken out from the device. Then, the height above ground was measured with a ruler, and the number of nodules was counted.

### Determination of fresh weight, dry weight, and chlorophyll content

Before the plants were harvested, the chlorophyll content of the third compound leaf of *S. rostrata* was measured by using a chlorophyll meter (SPAD502 Plus, Konica Minolta), and the average value of each single leaf was taken as the result. The aboveground and underground parts of the plants were separated, and the fresh weight of the plants was determined. The aboveground and underground parts of the separated plants were killed in an oven at 95°C for 15 min and dried to constant weight at 80°C. Then, the dry weight was measured.

### Determination of SOD and CAT activity

The leaf samples of *S. rostrata* were quick-frozen in liquid nitrogen and ground into powder. Phosphate buffer was added for vortex extraction for 5 min, centrifuged at 3500 rpm for 15 min at 4°C, and the supernatant was taken for testing. Enzyme activity was measured by using total superoxide dismutase (T-SOD) assay kit (hydroxylamine method) and catalase (CAT) assay kit (visible light) that produced by Nanjing Jiancheng Bioengineering Institute. Definition of CAT activity units: The amount of 1 μmol hydrogen peroxide catalyzed by per gram of tissue per second at 37°C was defined as a CAT activity unit. Definition of SOD activity units: The amount of SOD corresponding to 50% SOD inhibition per gram of tissue in 1 ml of reaction solution was defined as a SOD activity unit.

### Determination of MDA content

Leaf samples were prepared according to the above method, and the content of MDA was determined using the plant malondialdehyde (MDA) assay kit (colorimetric method) produced by Nanjing Jiancheng Bioengineering Institute.

### Determination of PRO content

Leaf tissue homogenate was prepared using the same method for CAT content determination, and the supernatant after centrifugation was detected using a proline assay kit (colorimetric) produced by Nanjing Jiancheng Bioengineering Institute.

### Statistical analysis

Data among different treatments were analyzed by the IBM SPSS Statistics 21: the Levene test was first performed. If the Levene test was significant, Welch’s ANOVA and Games–Howell *Post-hoc* were used; if the Levene test was not significant, Fisher’s ANOVA and least significant difference (LSD) *Post-hoc* (*p* < 0.05) were used.

## Results

### Effects of ORS571 inoculation on *S. rostrata* under salt stress

#### Morphological characters

**Figure 1:**
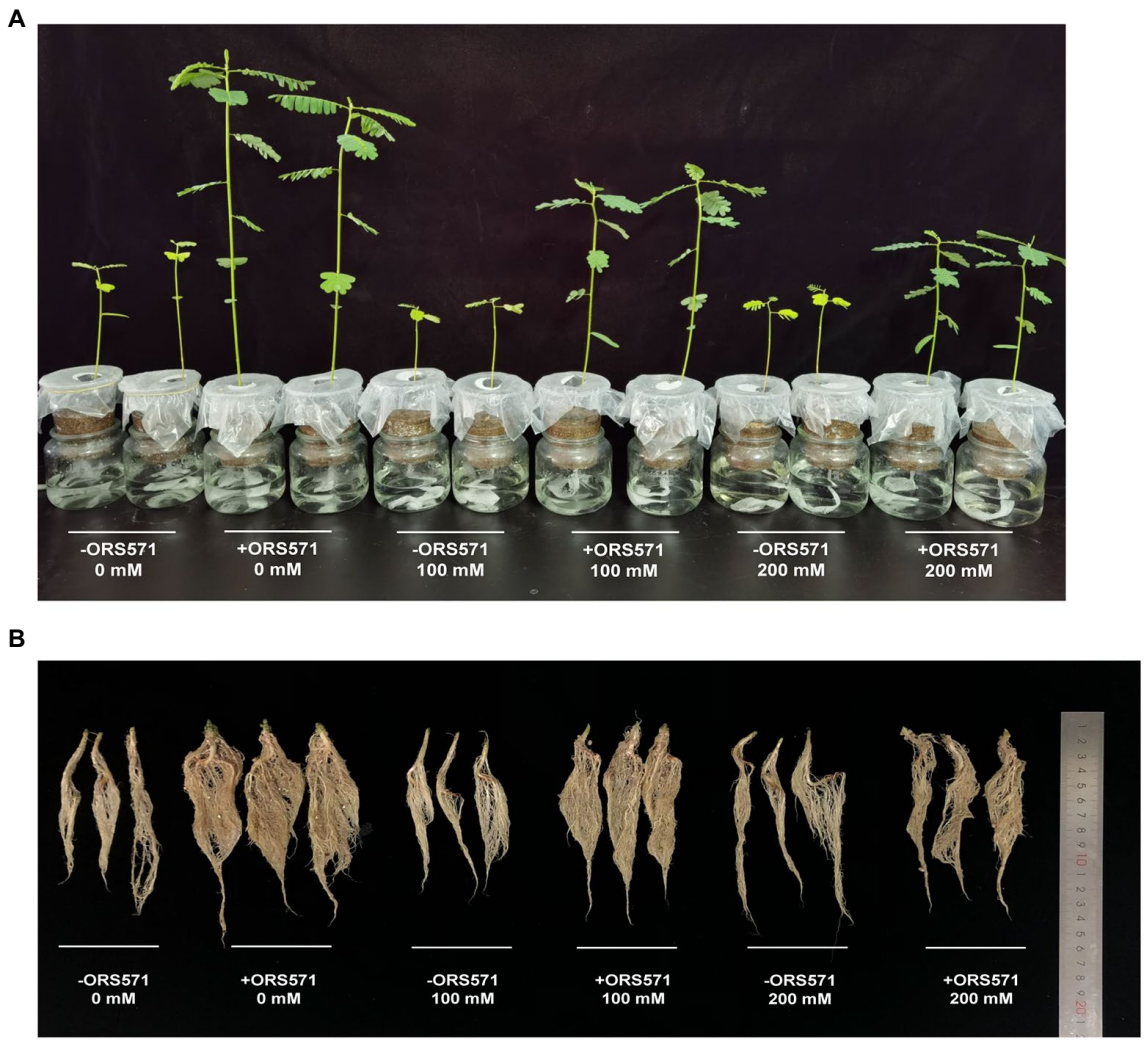
Representative images of *Sesbania rostrata* inoculated with ORS571 or uninoculated group as a control at different salt concentrations for 20 days. **(A)** Growth condition of the aboveground part. **(B)** Growth condition of the underground part.

**Figure 2:**
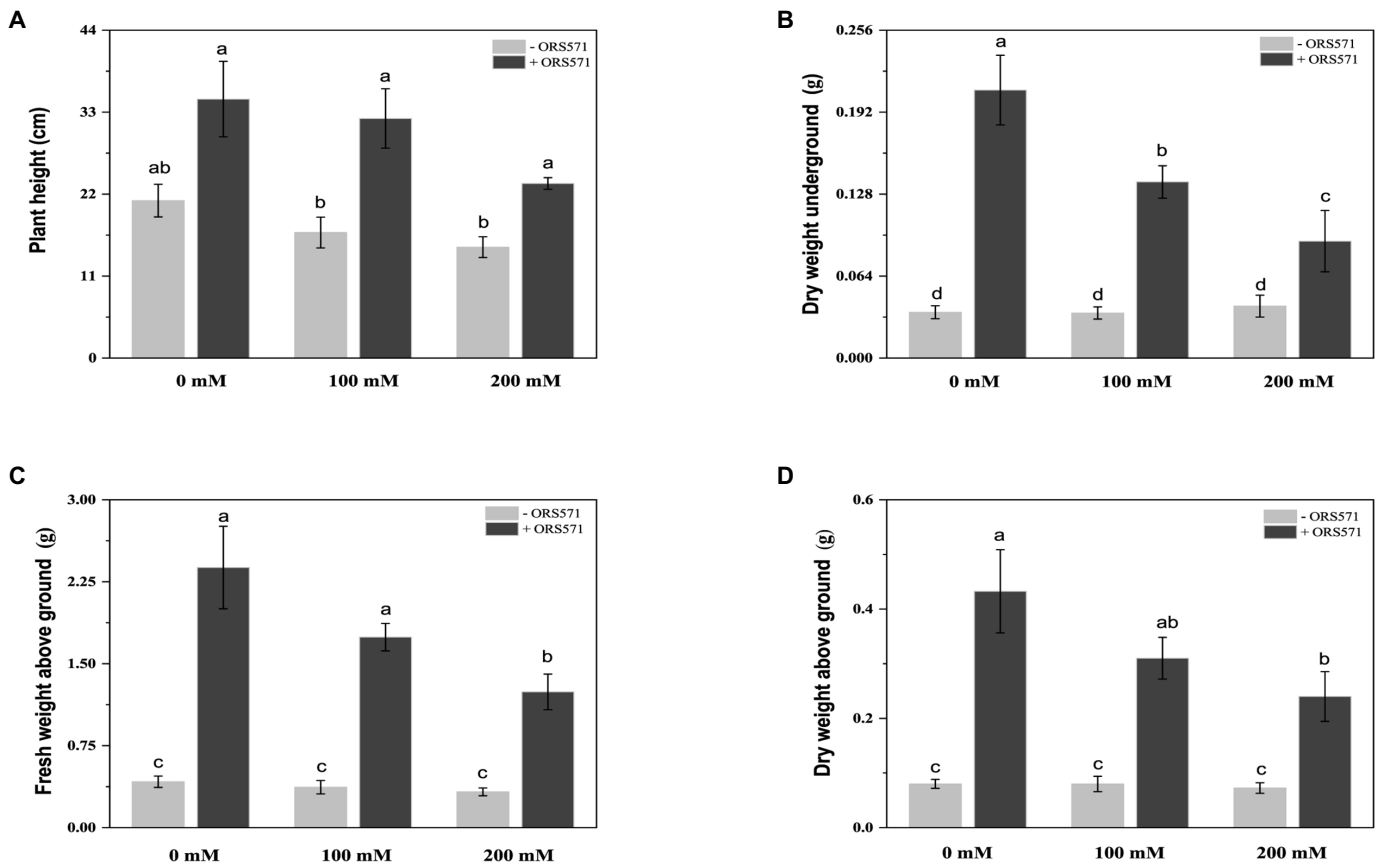
Effects of inoculation with ORS571 on the biomass of *Sesbania rostrata* under different salt concentrations. The plant height **(A)**, DW underground **(B)**, FW above ground **(C)**, DW above ground **(D)** were measured. Different lower-case letters in each column shape indicate a significant difference at *p* < 0.05 by statistical analysis. Error bars indicate standard deviations from four parallel replicates.

*S. rostrata* suffered significant salt damage during the 20 days of NaCl stress treatment. The height of the plant and the dry weight/fresh weight of aboveground tended to decrease with the increasing salt stress concentration (Figure 1). Inoculation with ORS571 significantly increased the height of the plant, the dry weight/fresh weight of aboveground and the dry weight of underground in both the stressed and non-stressed treatment groups (Figure 2). These results indicate that the ORS571 has a strong growth-promoting effect on *S. rostrata* and make the host better respond to the high salt concentrations.

#### Chlorophyll content and the nodulation number

**Figure 3:**
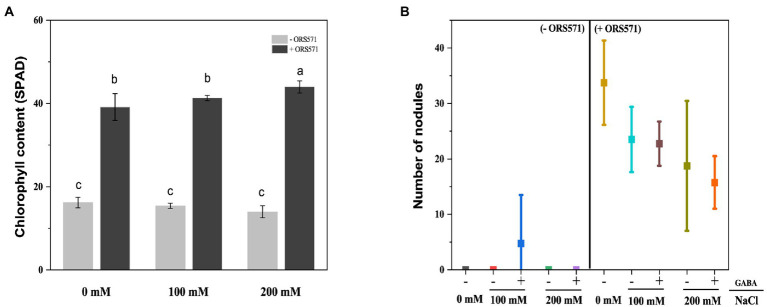
Chlorophyll content in leaf samples and nodule number inoculated with ORS571 or not under different salt concentrations. **(A)** The chlorophyll content of the inoculated group under salt stress was significantly higher than that of the uninoculated group. **(B)**The number of nodules formed by ORS571 was detected and decreased with increasing salt concentration. Different lower-case letters in each column shape indicate a significant difference at *p* < 0.05 by statistical analysis. Error bars indicate standard deviations from four parallel replicates.

The chlorophyll content in the leaves of *S. rostrata* was reduced under salt stress. After inoculation with ORS571, the chlorophyll content was significantly increased 2.42, 2.68, and 3.15 times compared with the uninoculated group (control) under 0, 100, and 200 mM salt stress, respectively (Figure 3A). In low-nitrogen and salt-stressed environment, the growth of *S. rostrata* was significantly impaired. The weak growth of *S. rostrata* did not respond well to salt stress in this environment. ORS571 inoculation significantly promoted the growth of *S. rostrata*. With the increase of salt concentration, the elevating effect of ORS571 on chlorophyll content was strengthened. This indicates that the rhizobia ORS571 is an important partner of *S. rostrata* to overcome the adverse environment. Additionally, the nodulation ability of ORS571 under salt stress was investigated. There were nodules on the roots of *S. rostrata* treated with ORS571, and the number of nodules decreased with increasing salt concentration (Figure 3B). This result suggests that salt stress impairs the symbiotic nodulation ability of ORS571, so ORS571 may play a role in enhancing the salt tolerance of *S. rostrata* in other aspects than just nitrogen fixation to promote growth.

#### Antioxidant enzyme activities, MDA content, and PRO content in leaves

SOD and CAT are the main enzymes in organisms that defend against oxidative damage. It can be seen from Table 1 that inoculation of ORS571 can significantly increase the CAT activity and SOD activity in leaves by 75 and 5% compared with the control under 100 mM NaCl, and it can significantly increase the CAT activity and SOD activity in leaves by 68 and 5% compared with the control under 200 mM salt stress. Under non-stress conditions, inoculation of ORS571 also significantly enhanced SOD and CAT enzyme activities. MDA content can reflect the degree of damage by abiotic stress which the plants suffered from. Compared with the normal situation, salt stress led to the increase of MDA level in the leaves of *S. rostrata* (Table 1). Under the conditions of 100 and 200 mM salt stress, ORS571 could significantly reduce the MDA content in leaves by 19 and 5%, respectively (Table 1). The above results indicate that ORS571 can indeed enhance the salt stress tolerance of *S. rostrata* by increasing the activity of antioxidant enzymes and reducing the content of MDA in leaves. PRO is one of the components of plant proteins that accumulates in the plants in response to environmental stress. As shown in Table 1, the accumulation of PRO in the leaves of *S. rostrata* increased with increasing salt concentration. Under 0, 100, and 200 mM salt stress, the PRO content in the leaves of *S. rostrata* following inoculation with ORS571 was increased by 43.7, 80, and 96.4%, respectively.

**Table 1:** Effect of ORS571 on CAT, SOD activity and MDA, PRO content in leaves under salt stress.

NaCl	ORS571	CAT activity U·g^−1^	SOD activity U·g^−1^	MDA content nmol·g^−1^	PRO content μg·g^−1^
0 mM	−	61.88 ± 1.66^e^	308.31 ± 8.87^c^	120.84 ± 5.98^c^	14.36 ± 0.51^e^
	+	165.8 ± 1.78^a^	315.83 ± 3.56^b^	127.49 ± 7.3^bc^	20.63 ± 0.70^d^
100 mM	−	51.94 ± 3.55^e^	309.36 ± 3.38^c^	145.09 ± 5.59^a^	18.67 ± 0.50^d^
	+	90.68 ± 1.81^c^	324.53 ± 1.74^a^	118.14 ± 5.62^c^	33.6 ± 2.89^b^
200 mM	−	77.01 ± 1.01^d^	310.26 ± 2.45^c^	141.17 ± 8.36^a^	26.61 ± 0.22^c^
	+	129.12 ± 0.94^b^	325.51 ± 1.91^a^	133.75 ± 3.24^bc^	52.25 ± 3.36^a^

Different lower-case letters in each column shape indicate a significant difference at *p* < 0.05 by statistical analysis.

### Effects of exogenous GABA on *S. rostrata* under salt stress

#### Morphological characters and chlorophyll content

**Figure 4:**
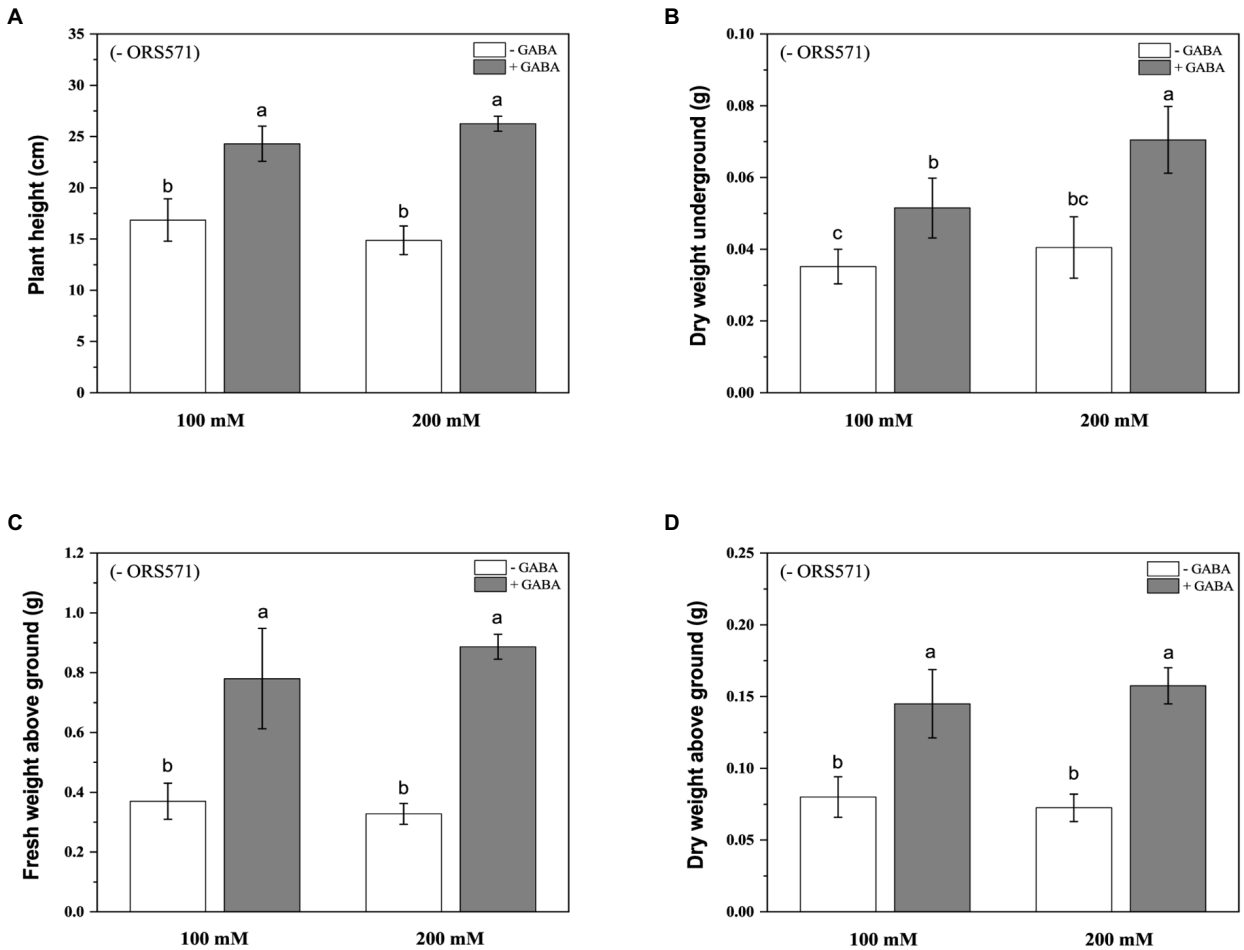
Effects of exogenous addition of GABA on the biomass aboveground/underground and plant height of *Sesbania rostrata* under different salt concentrations. The plant height **(A)**, DW underground **(B)**, FW above ground **(C)**, and DW above ground **(D)** were measured. Different lower-case letters in each column shape indicate a significant difference at *p* < 0.05 by statistical analysis. Error bars indicate standard deviations from four parallel replicates.

**Figure 5:**
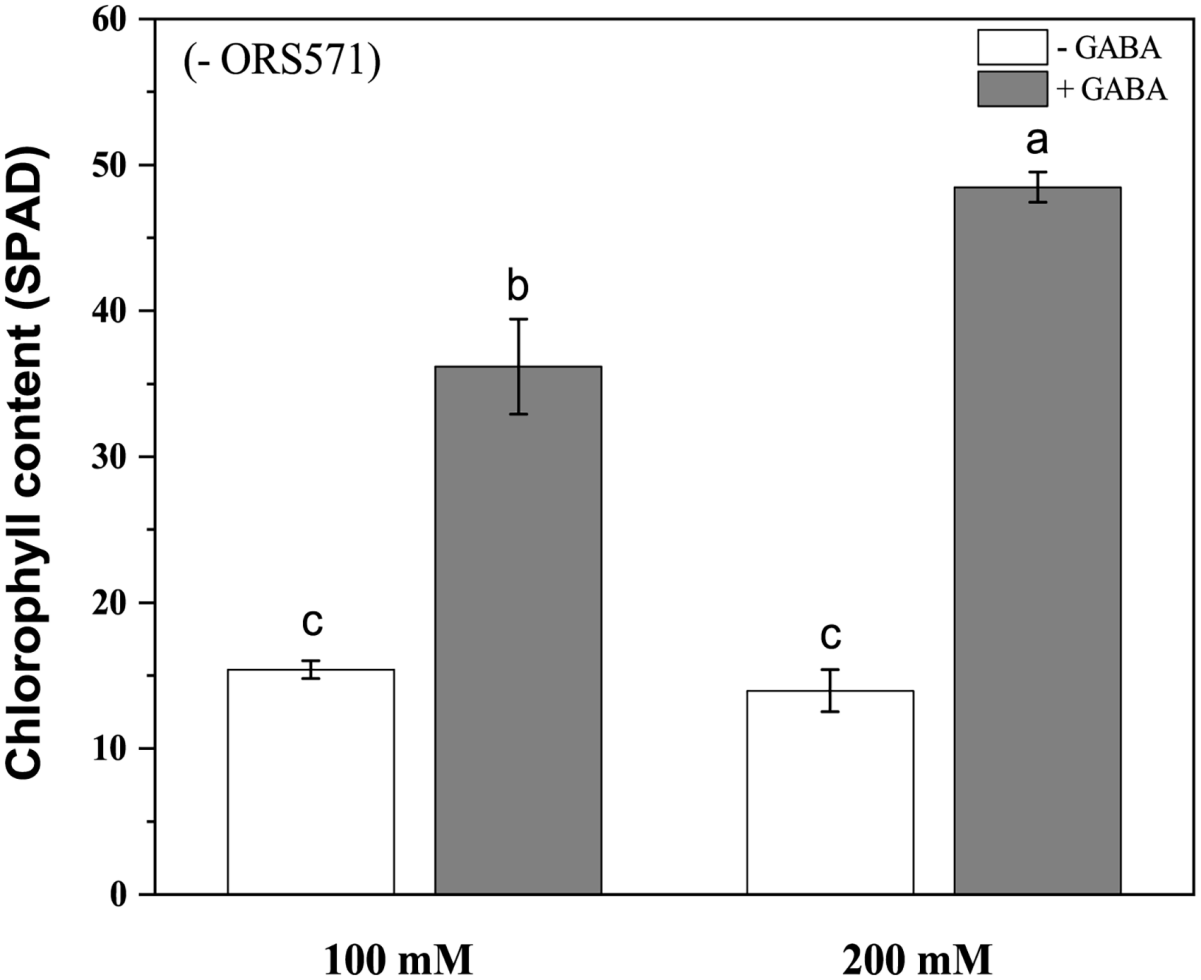
Chlorophyll content of *Sesbania rostrata* under NaCl stress with or without GABA. The chlorophyll content of GABA treatment was significantly higher than that of the control under salt stress. Different lower-case letters in each column shape indicate a significant difference at *p* < 0.05 by statistical analysis. Error bars indicate standard deviations from four parallel replicates.

In order to further improve the salt tolerance of *S. rostrata*, the study of exogenous addition of GABA was carried out. Under 100 mM salt stress, exogenous addition of GABA could significantly increase the plant height and biomass. Under 200 mm salt stress, exogenous addition of GABA significantly increased the plant height and aboveground/underground biomass (Figure 4). Moreover, during the salt stress period, the chlorophyll content of plant leaves in the GABA-treated group was significantly increased compared with the control group, which was increased by 2.35 and 3.47 times under 100 and 200 mM salt stress, respectively (Figure 5).

#### Antioxidant enzyme activities, MDA content, and PRO content in leaves

To reveal the relevant physiological mechanism that GABA treatment can enhance the salt tolerance ability of *S. rostrata*, several biochemical indicators were examined. Under 100 and 200 mM salt stress concentrations, the addition of GABA significantly increased the CAT activity in plant leaves by 74 and 46%, respectively, but there was no significant change in SOD activity. Under 100 mM salt stress, GABA treatment significantly decreased MDA content in leaves by 12%. It significantly decreased MDA content in leaves by 10.5% under 200 mM salt stress. In addition, the PRO content in leaves was significantly reduced by 28 and 27% following exogenous GABA treatment under 100 and 200 mM salt stress, respectively (Table 2).

**Table 2:** Effect of GABA on CAT, SOD activity and MDA, PRO content in leaves under salt stress.

NaCl	GABA mg·L^−1^	CAT activity U·g^−1^	SOD activity U·g^−1^	MDA content nmol·g^−1^	PRO content μg·g^−1^
100 mM	0	51.94 ± 3.55^d^	309.36 ± 3.38^b^	145.09 ± 5.59^a^	18.67 ± 0.50c
	200	90.38 ± 1.88^b^	307.85 ± 4.46^b^	128.91 ± 2.52^bc^	13.47 ± 0.35d
200 mM	0	77.01 ± 1.01^c^	310.26 ± 2.45^b^	141.17 ± 8.36^a^	26.61 ± 0.22a
	200	112.8 ± 5.48^a^	313.72 ± 0.93^ab^	126.32 ± 2.12^bc^	19.55 ± 2.91bc

Different lower-case letters in each column shape indicate a significant difference at *p* < 0.05 by statistical analysis.

### Effects of exogenous GABA on ORS571-*S. rostrata* symbiotic system under salt stress

#### Morphological characters and chlorophyll content

**Figure 6:**
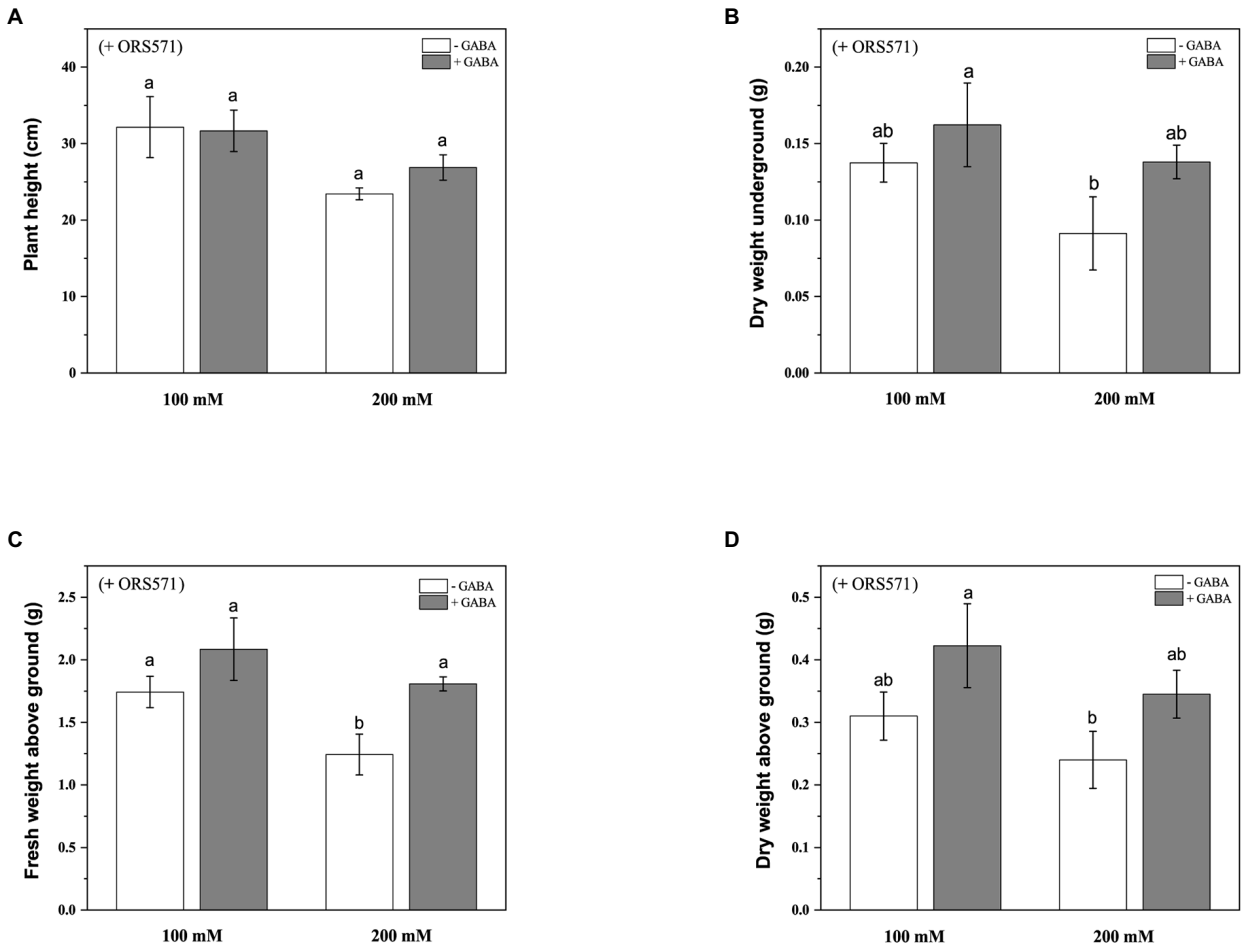
Effects of exogenous addition of GABA on the biomass aboveground/underground and plant height of ORS571*-Sesbania rostrata* symbiotic system under different salt concentrations. The plant height **(A)**, DW underground **(B)**, FW above ground **(C)**, and DW above ground **(D)** were measured. Different lower-case letters in each column shape indicate a significant difference at *p* < 0.05 by statistical analysis. Error bars indicate standard deviations from four parallel replicates.

**Figure 7:**
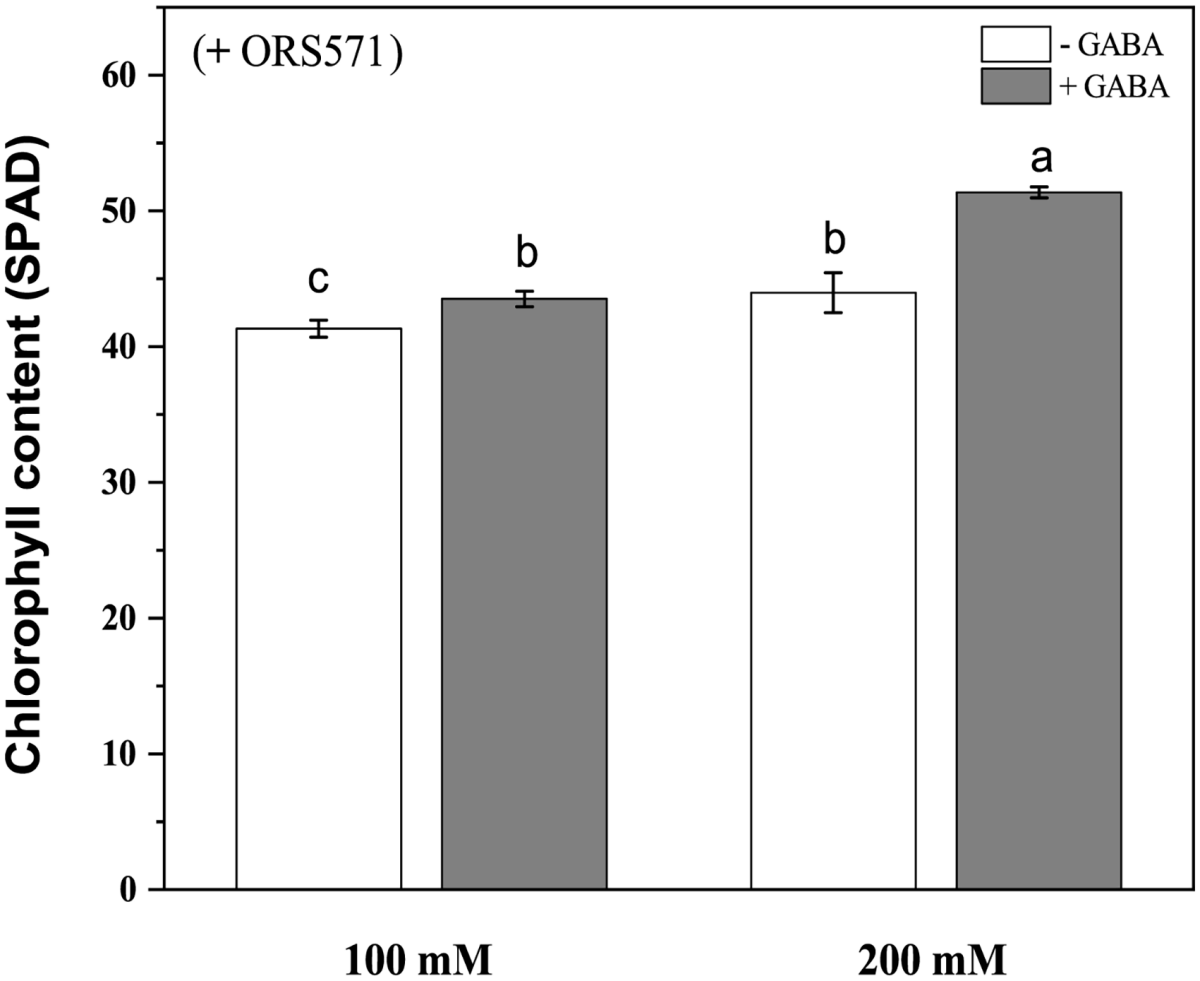
Chlorophyll content of *Sesbania rostrata* inoculated with ORS571 under NaCl stress with or without GABA. The chlorophyll content of GABA treatment was significantly higher than that of the control under salt stress. Different lower-case letters in each column shape indicate significant difference at *p* < 0.05 by statistical analysis. Error bars indicate standard deviations from four parallel replicates.

It was known from the above experiments that ORS571 had a strong ability of promoting growth and nitrogen fixation, and could improve salt tolerance of *S. rostrata*. In order to explore whether GABA and rhizobia have cooperative effects on improving the salt tolerance of *S. rostrata*, exogenous GABA was added to treat *S. rostrata* inoculated with ORS571, and the GABA-untreated group was used as a control. There was no significant effect of exogenous addition of GABA on the plant height and above/underground dry weight of ORS571-*S. rostrata* symbionts (Figures 6A,B,D). GABA treatment significantly promoted the fresh weight above ground in the ORS571*-S. rostrata* interaction system under 200 mM salt stress, while there was no significant change in the above/underground biomass under 100 mM salt stress (Figure 6C). This result may indicate that the interaction system of *S. rostrata-*Rhizobium can fully adapt to 100 mm salt concentration, which is no longer regarded as a stress factor. Under 100 and 200 mM salt stress, the chlorophyll content (43.5 ± 0.57 SPAD and 51.4 ± 0.40 SPAD, respectively) in the GABA-treated leaf of interaction system was significantly higher than that in the untreated group (41.3 ± 0.63 SPAD and 44 ± 1.46 SPAD, respectively; Figure 7).

#### Antioxidant enzyme activities, MDA content, and PRO content in leaves

The symbiosis of ORS571 with *S. rostrata* was able to significantly improve the plant salt tolerance. Exogenous addition of GABA further enhanced the ability of the interaction system to resist salt stress, so the relevant physiological mechanism was explored. Under 100 mM salt stress, the leaves CAT activity in the interaction system was significantly increased by 75% by exogenous addition of GABA, while the SOD activity and MDA content in leaves did not change significantly. Under 200 mM salt stress, exogenous addition of GABA significantly increased leaves CAT activity in the interaction system by 16%, and the MDA content in leaves was significantly reduced by 26%. In addition, there was no significant change in PRO content in ORS571-*S. rostrata* symbiosis system following exogenous GABA treatment under 100 mM salt stress. However, under 200 mM salt stress, the content of PRO in leaves of ORS571-*S. rostrata* symbiosis system was significantly decreased by 56% following exogenous GABA treatment (Table 3).

**Table 3:** Effect of GABA on CAT, SOD activity and MDA, PRO content in leaves of *Sesbania rostrata* inoculated with ORS571 under salt stress.

NaCl	ORS571	GABA mg·L^−1^	CAT activity U·g^−1^	SOD activity U·g^−1^	MDA content nmol·g^−1^	PRO content μg·g^−1^
100 mM	+	0	90.68 ± 1.81^c^	324.53 ± 1.74^a^	118.14 ± 5.62^b^	33.60 ± 2.89b
		200	158.3 ± 2.24^a^	324.37 ± 1.65^a^	117.62 ± 5.63^b^	33.72 ± 4.33b
200 mM	+	0	129.12 ± 0.94^b^	325.51 ± 1.91^a^	133.75 ± 3.24^ab^	52.25 ± 3.36a
		200	150.27 ± 2.58^a^	312.64 ± 3.21^bc^	98.48 ± 3.4^c^	22.75 ± 0.76c

Different lower-case letters in each column shape indicate a significant difference at *p* < 0.05 by statistical analysis.

## Discussion

Salt stress has serious negative effects on plant growth and development, mainly in terms of oxidative damage, ion disturbance, and osmotic imbalance (Hayashi and Murata, 1998). Salt-tolerant plants account for about 1% of all terrestrial plant species, mostly native to arid or semi-arid coastal areas, and can adapt to NaCl concentrations of up to 0.2–0.6 M (Kumari et al., 2015). *Sesbania* sp. has become a pioneer plant for improving coastal saline-alkali land due to its characteristics of salt tolerance, drought tolerance, waterlogging tolerance, high productivity, and adaptation to nutrient-poor habitats (Li et al., 2016). It has been shown that the addition of biochar or biochar-compost can effectively improve the barren saline soils and thus promote the growth of *Sesbania cannabina* (Luo et al., 2017). Another study confirmed that the combined application of microorganisms and biochar was effective in improving the tolerance to salt stress of *S. cannabina* (Cui et al., 2021). However, these optimizations of biological measures to improve salinized soils are far from adequate and more explorations are still needed. In the present study, we simulated a barren saline environment and found that inoculation of ORS571 could enhance the salt tolerance of *S. rostrata* by promoting the plant growth (Figures 1–3). Previous studies have shown that ROS induced by salt stress could lead to cytotoxicity, reduce the efficiency of the photosynthetic system, and even cause the death of plant cell (Apel and Hirt, 2004; Mittova et al., 2004; Sharma et al., 2012). It was also reported that inoculation of a PGPB, *Bacillus amyloliquefaciens* SOR9, could enhance the activity of antioxidants CAT and POD (Chen et al., 2016). The research of Jha and Bharti showed that inoculation with PGPB protected plants from the salt stress by increasing the expression and activity of antioxidant enzymes (Jha and Subramanian, 2014; Bharti et al., 2016). Consistent with the above studies, we further found that inoculation with ORS571 did increase the antioxidant enzyme activity as well as the stress tolerance of plants (Table 1). Previous studies showed that salt stress led to an increase in the lipid peroxidation product MDA, which was significantly decreased after inoculation with PGPB (Jha and Subramanian, 2014; El-Esawi et al., 2018; He et al., 2018; Sapre et al., 2018; Chauhan et al., 2019). Our study also confirmed these results. These results suggest that ORS571 stimulates the plant defense system to scavenge ROS under salt stress and has a direct effect on the alleviation of salt stress in *S. rostrata.* Plants respond and adapt to adversity stresses by accumulating PRO when subject to stresses such as drought, high salinity, high or low temperatures, and heavy metals (Szabados and Savouré, 2010; Kavi Kishor and Sreenivasulu, 2014). Studies have shown that rhizobia can change the amino acid composition of alfalfa under salt stress, thus playing a role in improving the salt tolerance of the symbiont (Bertrand et al., 2016). Consistent with the previous study, we observed that the content of PRO accumulated in *S. rostrata* under salt stress, and the content of PRO was significantly increased following inoculation with ORS571 under salt stress (Table 1). It is suggested that the growth-promoting effect of ORS571 may also lead to a significant increase in the content of amino acids in *S. rostrata*.

In addition, this study was not limited to inoculation of a single strain, but also attempted to find other ways to improve the tolerance to salt stress of *S. rostrata.* In recent years, GABA has been well known as a growth stimulator and signaling molecule to influence the plant stress tolerance both endogenously and exogenously (Shang et al., 2011; Malekzadeh et al., 2014; Wang et al., 2014b; Ramesh et al., 2015; Sheteiwy et al., 2019). Exogenous application of GABA has been shown to alleviate the inhibitory effects of salt stress on the plant growth by reducing chlorophyll degradation and maintaining high photosynthetic capacity (Shelp et al., 2017; Wu et al., 2020). Studies have shown that exogenous GABA treatment mitigates damage caused by salt stress by increasing growth rate, fresh and dry weight, and chlorophyll levels (Wang et al., 2014a; Wu et al., 2020). The above studies on exogenous application of GABA to improve salt tolerance in plants have mostly focused on general plants such as fruits and vegetables, but the present study was the first to use GABA to explore whether it could further improve salt tolerance in salt-tolerant plants, *S. rostrata*. Our results showed that exogenous addition of GABA under salt stress increased the plant height, biomass, and the chlorophyll content of *S. rostrata*, more significantly at high salt concentrations (200 mm), which is consistent with previous studies (Figures 4, 5). At the same time, the antioxidant capacity of *S. rostrata* was improved with the treatment of GABA under salt stress, which explained the mechanism of GABA alleviating salt stress physiologically. It is interesting that the PRO content of leaves in *S. rostrata* was significantly reduced following the addition of GABA under salt stress (Table 2). Generally, a strong correlation is established between PRO accumulation and abiotic stress tolerance in many plants (Hare and Cress, 1997; Siripornadulsil et al., 2002; Kavi Kishor et al., 2005; Verbruggen and Hermans, 2008). However, PRO accumulation does not seem to play a role in salt tolerance in barley, instead representing a symptom of salt-susceptibility (Chen et al., 2007; Widodo Patterson et al., 2009). Our results suggested that exogenous GABA may improve salt stress tolerance in *S. rostrata* without the need to accumulate excessive PRO in response to salt stress.

Studies have shown that various methods have been tried to alleviate the growth inhibition of *Sesbania* sp. in saline soil, such as the co-application of biochar and effective microorganisms (Cui et al., 2021). To maximize the salt stress tolerance of *S. rostrata*, inoculation with ORS571 and exogenous GABA were applied in combination. The experimental results showed that the fresh weight and chlorophyll content of *S. rostrata* inoculated with ORS571 were increased after GABA treatment under salt stress (Figures 6, 7), and further studies revealed an increase in antioxidant enzyme activity compared to the control (Table 3). This indicates that exogenous application of GABA can also scavenge ROS in the plant defense system under salt stress, and may also alleviate salt stress by preventing stress-induced damage to chloroplast structure. Moreover, exogenous addition of GABA to the ORS571-*S. rostrata* symbiont showed a consistent trend of PRO and MDA contents under salt stress. There was no significant change under 100 mM salt stress, and a significant decrease under 200 mM salt stress (Table 3). We speculated that 100 mm salt concentration did not cause greater stress on the ORS571-*S. rostrata* symbiont, so the levels of plant stress metabolites did not change significantly. In contrast, exogenous GABA treatment and inoculation with ORS571 exerted a cooperative effect to improve the salt tolerance of *S. rostrata* at 200 mM salt concentration, thus reducing the levels of plant stress metabolites MDA and PRO. The above results are consistent with the results of previous studies, and also means that GABA has a positive effect on the salt stress tolerance of *S. rostrata* inoculated with ORS571.

Initial studies on the mechanism of salt tolerance in *S. rostrata* showed that *S. rostrata* could translocate more Na^+^ and Cl^−^ ions from roots to shoots and trap them in the shoot cells compared with salt-sensitive plants (Jungklang et al., 2003). In addition, compared to salt-sensitive plants, higher constitutive or inducible antioxidant enzyme activities such as SOD and CAT also protected *S. rostrata* from ROS damage under NaCl stress (Jungklang et al., 2004). Our study confirmed that inoculation with ORS571 and exogenous administration of GABA could further increase the activity of antioxidant enzymes in *S. rostrata* and reduce the content of lipid peroxidation product MDA. This may be one of the mechanisms by which ORS571 and GABA enhanced the salt tolerance of *S. rostrata*. Moreover, we also speculated that inoculation with ORS571 and exogenous application of GABA might also accelerate the Na^+^ and Cl^−^ ions content from roots to shoots. On the other hand, studies have shown that endogenous GABA plays an important role in improving salt tolerance of plants, which is often induced by salt stress or exogenous GABA (Shelp et al., 2012, 2017). GAD genes involved in GABA metabolism in plants are sensitive to abiotic stress and their enzymatic activities are closely related to stress tolerance of plants (Akcay et al., 2012). Given that GAD genes have not been characterized in *S. rostrata*, we speculate that inoculation with ORS571 and exogenous application of GABA may induce changes in endogenous GABA metabolism. With subsequent genome sequencing of the *S. rostrata*, more molecular mechanisms will be excavated.

In conclusion, this study proposed a way to enhance the salt stress tolerance of *S. rostrata*, and revealed partial salt-tolerance mechanism. *S. rostrata* suffered from growth damage at higher salt concentrations. ORS571 may confer salt stress tolerance in *S. rostrata* by increasing chlorophyll synthesis, antioxidant enzyme activity, and proline content. Exogenous GABA may also confer salt stress tolerance in *S. rostrata* by increasing chlorophyll synthesis and antioxidant enzyme activity. Additionally, inoculation with ORS571 and exogenous application of GABA can exert a cooperative effect to enhance the salt tolerance of *S. rostrata*. This study will provide opportunities for increasing the salt tolerance of the green manure plant and improving saline land by biological engineering.

## Data availability statement

The original contributions presented in the study are included in the article/supplementary material; further inquiries can be directed to the corresponding author.

## Author contributions

ZX, YL, XL, and XD designed the research. YL and JY performed the experiments and analyzed the data. YL wrote the paper and contributed to analytical tools. All authors contributed to the article and approved the submitted version.

## Funding

This study was financially supported by the National Key Research and Development Program (2019YFD1002700), the National Natural Science Foundation of China (31870020), and by the Strategic Priority Research Program of the Chinese Academy of Sciences (XDA23050102).

## Conflict of interest

The authors declare that the research was conducted in the absence of any commercial or financial relationships that could be construed as a potential conflict of interest.

## Publisher’s note

All claims expressed in this article are solely those of the authors and do not necessarily represent those of their affiliated organizations, or those of the publisher, the editors and the reviewers. Any product that may be evaluated in this article, or claim that may be made by its manufacturer, is not guaranteed or endorsed by the publisher.
